# Temporal aiming

**DOI:** 10.1038/s41377-020-00360-1

**Published:** 2020-07-20

**Authors:** Victor Pacheco-Peña, Nader Engheta

**Affiliations:** 1 School of Mathematics, Statistics and Physics, Newcastle University, Newcastle Upon Tyne, NE1 7RU UK; 2 Department of Electrical and Systems Engineering, University of Pennsylvania, Philadelphia, PA 19104 USA

**Keywords:** Metamaterials, Nanophotonics and plasmonics, Nanophotonics and plasmonics

## Abstract

Deflecting and changing the direction of propagation of electromagnetic waves are needed in multiple applications, such as in lens–antenna systems, point-to-point communications and radars. In this realm, metamaterials have been demonstrated to be great candidates for controlling wave propagation and wave–matter interactions by offering manipulation of their electromagnetic properties at will. They have been studied mainly in the frequency domain, but their temporal manipulation has become a topic of great interest during the past few years in the design of spatiotemporally modulated artificial media. In this work, we propose an idea for changing the direction of the energy propagation of electromagnetic waves by using time-dependent metamaterials, the permittivity of which is rapidly changed from isotropic to anisotropic values, an approach that we call *temporal aiming*. In so doing, here, we show how the direction of the Poynting vector becomes different from that of the wavenumber. Several scenarios are analytically and numerically evaluated, such as plane waves under oblique incidence and Gaussian beams, demonstrating how proper engineering of the isotropic—anisotropic temporal function of ε_r_(t) can lead to a redirection of waves to different spatial locations in real time.

## Metamaterials: temporal beam steering

Theoretical analysis shows that beam-steering of electromagnetic waves can be accomplished by temporally changing the permittivity of metamaterials between isotropic to anisotropic values. The approach, called “temporal aiming”, has been formulated by Victor Pacheco-Peña from Newcastle University, UK and Nader Engheta from University of Pennsylvania in the US. In principle, it could open new opportunities for the flow of information around integrated photonic circuits, with flat metamaterial elements deflecting electromagnetic waves to specific targets or receivers on an optical chip as desired. Simulations performed with the software COMSOL with both plane, monochromatic waves and more complex Gaussian beams confirm the feasibility of the approach. It is proposed that the required temporal changes in the metamaterial’s relative permittivity could be achieved by the use of tunable metasurfaces or transmission lines with time-varying circuit elements.

## Introduction

Achieving arbitrary control of electromagnetic wave propagation has been of great interest within the scientific community for many years. It is well known that carefully engineered spatially varying geometries and materials can be implemented to manipulate wave–matter interactions^1^. This spatial control of waves is, in fact, the mechanism behind the development of many applications we often use on a regular basis, such as lenses, sensors and radars. The field of antennas has also benefited from this spatial control of wave propagation, where, for instance, in its basic configuration, it is possible to change the direction of a transmitted wave by mechanically modifying the spatial location of the transmitter, a technique known as *mechanical beam steering*^2^.

To further improve the spatial control of waves and to manipulate wave–matter interactions at will, metamaterials (and metasurfaces as their two-dimensional (2D) version) have been proposed in recent decades^3,4^. They have been demonstrated to provide engineering of their electromagnetic parameters, such as the permittivity (*ε*) and permeability (*µ*), achieving extreme parameter values that are, e.g., negative^5–8^ or near-zero^9–14^. They have been studied and demonstrated in different wave mechanisms and spectral bands ranging from acoustics, microwave and millimetre waves, terahertz waves and optics^15–19^. The freedom offered by metamaterials and metasurfaces, along with their compact designs, has opened up new avenues to improve the performance of devices and to develop new technologies, such as mathematical operators^20^, antennas and lenses^21–24^, sensors^25–27^ and polarisation converters^28–30^.

The *beam steering* of electromagnetic waves has also benefited from the introduction of metamaterials, where it has been shown that electromagnetic radiated beams can be redirected by changing the position of the source in a metalens–antenna system, by locally designing the phase of the unit cells in the metamaterials, or by real-time tuning of the effective electromagnetic parameters of the metastructures^31–36^, among other techniques. These steering properties are important in different areas, such as in point-to-point communications and radars, where the spatial aiming of targets is required.

Metamaterials and metasurfaces have so far been studied mostly in the time-harmonic scenario, where wave propagation is controlled by engineering geometries and materials in the spatial region, i.e., spatial inhomogeneity, in which the wave is travelling. Recently, the temporal modulation of metamaterials has also gained growing attention within the scientific community^37–44^, as changing the electromagnetic properties (*ε*, *µ*) of metamaterials both in space (*x, y, z*) and time (*t*) can offer full four-dimensional spatiotemporal control of wave–matter interactions. It is important to highlight that the interaction of electromagnetic waves in a time-modulated medium has been of great interest in the scientific community for several decades, where, for instance, in the last century, it was considered a time-dependent relative permittivity *ε*_*r*_(*t*) that is rapidly changed in time from one positive value *ε*_*r*1_ (greater than unity) to a different greater-than-unity positive value *ε*_*r*2_^40,41^. With this configuration, it was demonstrated that a set of two waves is created at this temporal boundary, one of which is travelling forward (FW), and the other, backward (BW). Remarkably, an analogy between this temporal and the spatial interface between two materials with different electromagnetic parameters was demonstrated, showing that the two waves created at a temporal boundary (FW and BW) are the temporal equivalent/analogy to the transmitted and reflected waves in spatial interfaces. In this realm, temporal and spatiotemporal metamaterials have recently been proposed and applied in several exciting and intriguing applications, such as effective medium theory^45^, inverse prisms^46^, nonreciprocity^47,48^, anti-reflection temporal coatings^49^, frequency conversion^50^ and time reversal^51^.

Motivated by the exciting possibilities and opportunities opened up by the spatiotemporal modulation of metamaterials in four dimensions (*x, y, z, t*) and the importance of the beam steering of electromagnetic waves for different applications, in this work, we introduce the concept of temporal aiming as the temporal analogue of spatial aiming. First, the fundamental physics of the proposed technique are presented, considering an oblique incident *p-*polarised monochromatic plane wave propagating in an unbounded medium with a time-dependent permittivity *ε*_*r*_(*t*). A temporal interface is introduced by changing the relative permittivity *ε*_*r*_(*t*) from an isotropic positive value *ε*_*r*1_ to an anisotropic permittivity $\overline{\overline {\varepsilon _{r2}}}$ = {*ε*_*r*2*x*_, *ε*_*r*2*z*_} (all with positive values greater than unity) at time *t* = *t*_1_. In so doing, the wavenumber ***k*** is preserved before and after the temporal change, but the direction of the energy (Poynting vector ***S***) is modified to a different angle compared with ***k***. The dependence of the new direction of the Poynting vector on the incident angle before the temporal change of *ε* and the values of the permittivity tensor $\overline{\overline {\varepsilon _{r2}}}$ is presented and discussed. Next, we study more complex scenarios by using Gaussian beams under normal and oblique incidence to consider the case of multiple plane waves travelling in different directions. With this set-up, it is demonstrated how the direction of the energy is modified to multiple angles when using an isotropic-to-anisotropic temporal boundary. Finally, the temporal aiming technique is numerically demonstrated by using a narrowband wavepacket under oblique incidence. As will be shown, this transmitted wavepacket can be “ushered” and “re-directed” in real time to reach different receivers placed at different spatial locations by properly engineering the *ε*_*r*_(*t*) of the background medium. All the results presented here are compared with numerical simulations, demonstrating good agreement with the design and analytical calculations.

## Results

### Temporal aiming: isotropic-to-anisotropic change in *ε*_*r*_

**Fig. 1:**
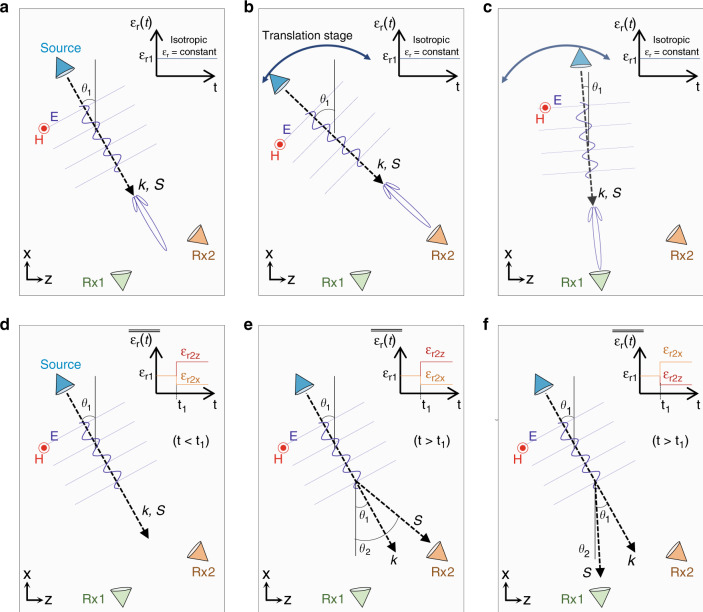
Schematic representation and comparison of spatial and temporal aiming. **a**–**c** Sketch of the conventional mechanical beam-steering technique, where the beam emitted by a source can be directed to either receiver 1 (Rx1) or receiver 2 (Rx2) by simply placing the source on a translation stage and moving it on the *xz* plane. **d**–**f** Temporal aiming sketch, considering a source emitting a monochromatic wavepacket embedded in a time-dependent medium, where its relative permittivity is changed from an isotropic value *ε*_*r1*_ to a tensor $\overline{\overline {\varepsilon _{r2}}}$*=* [*ε*_*r*2*x*_*, ε*_*r*2*z*_] at *t* = *t*_1_. Vectors ***S*** and ***k*** are parallel before the change in *ε*_*r*_ for *t* < *t*_1_, **d**, and non-parallel for *t* > *t*_1_, **e**–**f**. The angle of the vector ***S*** can be designed to reach either **e**, receiver 2 or **f**, receiver 1, depending on the tensor $\overline{\overline {\varepsilon _{r2}}}$ = [*ε*_*r*2*x*_*, ε*_*r*2*z*_]

First, let us discuss the spatial scenario schematically described in Fig. 1a. In this case, we can consider a monochromatic continuous wave (CW) that is being emitted by a source located on the *xz* plane and tilted to an angle *θ*_*1*_. The background medium is homogeneous, isotropic and time-independent, with a relative permeability *µ*_*r*_(*t*) = *µ*_*r*1_ and relative permittivity *ε*_*r*_(*t*) = *ε*_r1_ (see the inset of Fig. 1a). As is well known, the wave emitted from this source will also be tilted with the wavenumber ***k*** and Poynting vector ***S*** parallel, travelling along the same direction defined by *θ*_1_. Now, let us place two receivers at two different spatial locations (Rx1 and Rx2), as schematically shown in Fig. 1a. As observed, this is not the best scenario if one needs to send the emitted wave to either of the two receivers since the source is not aligned to Rx1 or to Rx2. However, as described in the introduction, the most simple yet effective way to reach either Rx1 or Rx2 is to perform mechanical beam steering^2,32^. In this technique, the source is placed on a translation stage, and then spatially shifted to different locations on the *xz* plane to tilt the emitted wave to the correct angle *θ*_*1*_ such that it reaches either Rx2 (Fig. 1b) or Rx1 (Fig. 1c). In addition to this technique, there are other effective and more sophisticated alternatives that can be used to steer electromagnetic waves, such as phased arrays (in which the phase shifts between the antenna elements can be changed in real time) and metamaterial-based antennas with tuneable properties^31,34,36^. In this context, steering electromagnetic waves is considered a key feature in applications where the spatial aiming of targets is needed, such as radars and point-to-point communications, as explained before.

The *spatial aiming* described in Fig. 1a–c has been discussed considering the time-harmonic scenario (frequency domain), taking into account that the relative permittivity and permeability of the background medium are constant [*ε*_*r*_(*t*) = *ε*_*r*1_, *µ*_*r*_(*t*) = *µ*_*r*1_]. With this in mind, one may ask the following: would it be possible to change the direction of the energy propagation of an emitted monochromatic CW plane wave in time by considering a time-dependent permittivity *ε*_*r*_(*t*) ≠ *ε*_*r*1_ of the background medium? If such temporal aiming is possible, what type of function for *ε*_*r*_(*t*) may we use to achieve it? To answer these questions, in this work, we focus our attention on the rapid change in relative permittivity (approximately modelled mathematically as a “step function”), which is rapidly modified in time from an initial value (equal to or greater than unity) ε_r1_ to another greater-than-unity *ε*_*r*2_ at a time *t* = *t*_1_, considering that the fall/rise time is smaller than the period T of the incident wave. (Strictly speaking, one needs to take into account the material dispersion in these scenarios. However, if we assume that the material resonance frequencies are much larger than the frequency of operation, we can approximately assume the materials to be dispersionless).

Let us study the case schematically shown in Fig. 1d. We again assume a *p-*polarised monochromatic CW plane wave travelling in an unbounded medium with an incident angle *θ*_*1*_. Let us first consider that the permittivity is time-dependent *ε*_*r*_(*t*) and is isotopically changed from a positive value *ε*_*r*1_ to another positive value *ε*_*r*2_. This scenario was studied in the last century, and it was shown that this *ε*_*r*_(*t*) can induce a temporal boundary/interface that generates a FW (temporal transmission) and a BW (temporal reflection) wave travelling with the same angle as that of the incoming wave, i.e., the wavenumber ***k*** is preserved while the frequency is changed from *f*_1_ to *f*_2_ = ($\sqrt {\varepsilon _{r1}}$/$\sqrt {\varepsilon _{r2}}$)*f*_1_. This type of time-dependent *ε*_*r*_(*t*) was then recently used to propose exciting applications, such as time reversal and anti-reflection temporal coatings, as explained in the introduction. Nevertheless, since ***k*** is the same before (*t* = *t*_1_^−^) and after (*t* = *t*_1_^+^) the change in *ε* from *ε*_*r*1_ to *ε*_*r*2_ and the direction of propagation for both ***k*** and energy ***S*** is not modified, it is straightforward to conclude that this type of *ε*_*r*_(*t*) is not suitable for the temporal aiming described in Fig. 1, since our aim is to redirect the energy of emitted waves to different receivers placed at different spatial locations.

Now, what if we change the relative permittivity ε_r_ from an isotropic value *ε*_*r*1_ to an anisotropic permittivity tensor at *t* = *t*_1_ such that $\overline{\overline {\varepsilon _{r2}}}$ = [*ε*_*r*2*x*_*, ε*_*r*2*z*_] with *ε*_*r*2*x*_ ≠ *ε*_*r*2*z*_, both of which are positive, real-valued and greater-than-unity parameters? The schematic representation of this *ε*(*t*) is shown in the inset of Fig. 1d. Note that here, we consider only the *z* and *x* components of the permittivity tensor since we have a TM polarisation with the electric field lying on the *xz* plane. Akbarzadeh et al. recently explored this function of *ε*_*r*_(*t*) to create what they aptly called the “inverse prisms”, demonstrating that vector ***k*** is again preserved as the isotropic case while the frequency is modified, but this time with a value depending on the tensor $\overline{\overline {\varepsilon _{r2}}}$ and the incident angle *θ*_*1*_^46^, hence their coined name, i.e., “inverse prism”. However, one may ask an intriguing question: what will happen to the Poynting vector ***S*** in this scenario? Would it be possible to exploit this isotropic-to-anisotropic temporal variation of ε(t) for temporal aiming, as we propose here?

To answer these questions, let us analytically evaluate this case, as shown in Fig. 1d. For *t* < *t*_1_, the background medium is isotropic and homogeneous with relative permittivity and relative permeability values ε_r1_ and µ_r1_, respectively. With this set-up (assuming the *e*^(*iωt*)^ time convention), the magnetic and electric fields are $H_1 = \hat ye^{i\left( {\omega _1t - k_xx - k_zz} \right)}$ and $$E_1 = \frac{1}{{\varepsilon _{0}{\omega_1}\varepsilon _{r1}}}e^{i\left( {\omega _1t - k_xx - k_zz} \right)}\left[ {\begin{array}{*{20}{c}} {k_z} & 0 & { - k_x} \end{array}} \right]$$, respectively, with ω_1_ = 2π*f*_*1*_, *k*_*x*_ = –*k*cos*(θ*_*1*_*), k*_*z*_ = *k*sin*(θ*_*1*_*)*, $k = \omega _1/v_1$, $v_1 = c/\sqrt {\mu _{r1}\varepsilon _{r1}}$ and *c* being the velocity of light in vacuum. At *t* = *t*_1_, the relative permittivity is changed to $\overline{\overline {\varepsilon _{r2}}}$ = [*ε*_*r*2*x*_, *ε*_*r*2*z*_], and for completeness, the permeability is changed to *µ*_*r*2*y*_. As mentioned before, this temporal boundary creates a set of FW (E_2_^+^, H_2_^+^) and BW (E_2_^−^, H_2_^−^) waves, with the total electric and magnetic fields defined as E_2_ = E_2_^+^ + E_2_^−^ and H_2_ = H_2_^+^– H_2_^−^, respectively. After applying the temporal boundary conditions for vectors **B** and **D** at the temporal boundary (**D**_**t1-δ**_ = **D**_**t1+δ**_ and **B**_**t1-δ**_ = **B**_**t1+δ**_ in the limit when δ→0^43^), it is straightforward^46^ to calculate the normalised amplitude of the electric field for both FW and BW waves (for the sake of completeness and easy access, we also give the complete derivation of the fields in Supplementary Materials section 1), resulting in the following expressions:

1 $$\frac{{E_2^ \pm }}{{E_1}} = \frac{1}{2}\left[ {\frac{{\mu _{r2y}{\omega_2} \pm \mu _{r1}{\omega_1}}}{{\mu _{r2y}{\omega_2}}}} \right]\frac{{\varepsilon _{r1}\sqrt {\varepsilon _{r2z}^2k_z^2 + \varepsilon _{r2x}^2k_x^2} }}{{\varepsilon _{r2x}\varepsilon _{r2z}\sqrt {k_z^2 + k_x^2} }}$$

with $\omega _2 = c\sqrt {[{k_x^2/({\varepsilon _{r2z}\mu _{r2y}})}] + [{k_z^2/( {\varepsilon _{r2x}\mu _{r2y}})}]}$. As observed, the new frequency ω_2_ and the amplitude of the generated FW and BW waves clearly depend on the incident angle *θ*_*1*_ and the values of *µ*_*r*2*y*_ and tensor $\overline{\overline {\varepsilon _{r2}}}$ = [*ε*_*r*2*x*_, *ε*_*r*2*z*_], as expected. Note that Eq. (1) reduces to the one shown in ref. ^46^ when *µ*_*r*2*y*_ = *µ*_*r*1_. Moreover, in the special case where the change in permittivity/permeability is isotropic (*ε*_*r*2_ = *ε*_*r*2*x*_ = *ε*_*r*2*z*_, *µ*_*r*2_ = *µ*_*r*2*y*_), Eq. (1) is reduced to the case described in refs. ^40,41^ with $\left( {E_2^ \pm /E_1} \right) = 0.5\left[ {\left( {\varepsilon _{r1}/\varepsilon _{r2}} \right) \pm \left( {\sqrt {\mu _{r1}\varepsilon _{r1}} /\sqrt {\mu _{r2}\varepsilon _{r2}} } \right)} \right]$.

Equation (1) denotes the amplitude of the FW and BW waves; however, it is important to remark that each component along the *x* and *z* axes (E_2×_^+^, E_2z_^+^, E_2×_^−^, E_2z_^−^) will be differently affected depending on the values of $\overline{\overline {\varepsilon _{r2}}}$ = [*ε*_*r*2*x*_, *ε*_*r*2*z*_] (the complete expressions for each component can be found in Supplementary Materials section 1). In this context, since we have a *p*-polarised monochromatic CW wave, it is interesting to evaluate the behaviour of the Poynting vector ***S***. From the derivation shown in Supplementary Materials section 1, it can be demonstrated that for times *t* > *t*_1_^+^, the momentum ***k*** is preserved (*θ*_*1k*_ = *θ*_*2k*_ = *θ*_*1*_), while the direction of the Poynting vector for both FW and BW waves can be calculated as $\theta _{2S} = \theta _{{\mathrm{SFW}}} = \theta _{{\mathrm{SBW}}} = {\mathrm{tan}}^{ - 1}\left( { - E_{2x}^ + /E_{2z}^ + } \right) = {\mathrm{tan}}^{ - 1}\left( { - E_{2x}^ - /E_{2z}^ - } \right)$, which can be reduced to the following simple expression:

2 $$\theta _{2S} = {\mathrm{tan}}^{ - 1}\left[ {\tan \left( {\theta _1} \right)\left( {\frac{{\varepsilon _{r2z}}}{{\varepsilon _{r2x}}}} \right)} \right]$$

From the expression above, one may notice that the direction of the energy flow is now different from the direction of the phase variation [*θ*_*2S*_ ≠ (*θ*_*2k*_ = *θ*_*1*_)], with the former angle depending on the angle of the incident wave before the temporal change (*θ*_*1*_) and the values of the relative permittivity tensor *ε*_*r*__2__*x*_ and *ε*_*r*__2__*z*_. A schematic representation of this performance is shown in Fig. 1e, f, where it is shown how the direction of the energy flow (***S***) is different from the direction of the wavenumber (***k***), and that the former can be steered in time by changing the relative permittivity from isotropic to anisotropic tensorial values, reaching the receivers Rx1 or Rx2, depending on the values of $\overline{\overline {\varepsilon _{r2}}}$ = [*ε*_*r*2*x*_*, ε*_*r*2*z*_] and the incident angle. In the following sections, we discuss analytical and numerical calculations using this temporal change of *ε*_*r*_ for plane waves and Gaussian beams to achieve temporal aiming with time-dependent metamaterials.

### Oblique incident plane wave: results obtained using isotropic-to-anisotropic *ε*(*t*)

**Fig. 2:**
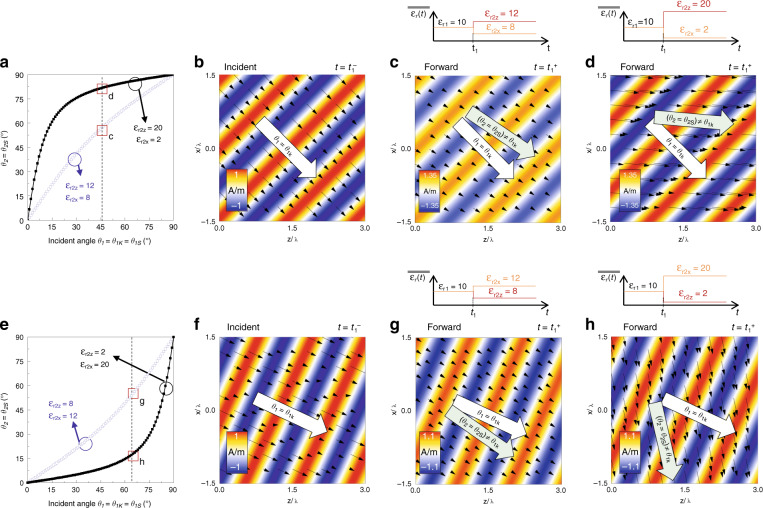
Analytical results of temporal aiming using plane waves. **a** Analytically derived angles of the instantaneous Poynting vector *θ*_2*s*_ (*t* > *t*_1_) as a function of the incident angle *θ*_*1*_ (*t* < *t*_1_) when *ε*_*r*_ is changed from isotropic *ε*_*r1*_ = 10 to anisotropic $\overline{\overline {\varepsilon _{r2}}}$ = [*ε*_*r*2*x*_ = 8*, ε*_*r*2*z*_ = 12] (blue circles) and to $\overline{\overline {\varepsilon _{r2}}}$ = [*ε*_*r*2*x*_ = 2*, ε*_*r*2*z*_ = 20] (black circles). **b** Snapshot of the H_y_ field (colour plot) and instantaneous Poynting vector (black arrows) distributions for an incident wave with *θ*_1_ = 45*°* at *t* < *t*_1_. **c**, **d** Snapshot of the H_y_ field of the FW wave (colour plot) and instantaneous Poynting vector (black arrows) distributions at *t* > *t*_1_ when *ε*_*r*_ is changed from *ε*_*r*1_ = 10 to $\overline{\overline {\varepsilon _{r2}}}$ = [*ε*_*r*2*x*_ = 8*, ε*_*r*2*z*_ = 12] and to $\overline{\overline {\varepsilon _{r2}}}$ = [*ε*_*r*2*x*_ = 2*, ε*_*r*2*z*_ = 20], respectively, for a time *t* = 38.2 *T* > *t*_1_. **e**, The same as in panel **a** but when *ε*_*r*_ is changed from *ε*_*r*1_ = 10 to $\overline{\overline {\varepsilon _{r2}}}$ = [*ε*_*r*2*x*_ = 12*, ε*_*r2z*_ = 8] (blue circles) and to $\overline{\overline {\varepsilon _{r2}}}$ = [*ε*_*r2x*_ = 20*, ε*_*r*2*z*_ = 2] (black circles). **f** The same as in panel **b** but with an incident angle *θ*_*1*_ = 65*°* at *t* < *t*_1_. **g**, **h** The same as in panels **c**, **d** but when *ε*_*r*_ is changed from *ε*_*r*1_ = 10 to $\overline{\overline {\varepsilon _{r2}}}$ = [*ε*_*r*2*x*_ = 12*, ε*_*r*2*z*_ = 8] and to $\overline{\overline {\varepsilon _{r2}}}$ = [*ε*_*r*2*x*_ = 20*, ε*_*r*2*z*_ = 2], respectively, for the same incident angle as in panel **f**, ***θ***_1_ = 65°. The time-dependent relative permittivity *ε*_*r*_*(t)* for the cases under study is shown at the top of panels **c**, **d** and **g**, **h**

Let us now evaluate the response of the proposed *temporal aiming* approach using a time-dependent ε_r_(t) that is rapidly changed from isotropic to anisotropic values. Without loss of generality, we consider a constant permeability (*µ*_*r*1_ = *µ*_*r*2_ = 1). The isotropic relative permittivity is initially *ε*_*r*1_ = 10 and is modified to $\overline{\overline {\varepsilon _{r2}}}$ at *t* = *t*_1_ = 38 *T*, where *T* is the period of the monochromatic incident wave before the change in permittivity occurs. Let us first use two different values for the relative permittivity tensor, i.e., $\overline{\overline {\varepsilon _{r2}}}$ = [*ε*_*r*2*x*_ = 8, *ε*_*r*2*z*_ = 12] and $\overline{\overline {\varepsilon _2}}$ = [*ε*_*r*2*x*_ = 2, *ε*_*r*2*z*_ = 20], noting that in both cases, *ε*_*r*2*z*_ > *ε*_*r*2*x*_. With this set-up, the analytical calculations of the angle of the Poynting vector *θ*_2*S*_ for *t* > *t*_1_ as a function of the incident angle *θ*_*1*_ using Eq. (2) are shown as blue and black circles in Fig. 2a. In this figure, it is clear how *θ*_2*S*_ depends on the tensor $\overline{\overline {\varepsilon _{r2}}}$ and *θ*_*1*_, as explained in the last section. Moreover, note that for the case with $\overline{\overline {\varepsilon _{r2}}}$ = [*ε*_*r2x*_ = 2, *ε*_*r*2*z*_ = 20], *θ*_2*S*_ is larger for smaller values of *θ*_*1*_ than in the case with $\overline{\overline {\varepsilon _{r2}}}$ = [*ε*_*r*2*x*_ = 8, *ε*_*r*2*z*_ = 12]. For instance, if *θ*_1_ = 15°, then *θ*_2*S*_ will be 69.5° and 21.9° for each case, respectively. These results are expected since the amplitude of the *x* and *z* components of the electric field for *t* > *t*_1_ can be increased or reduced depending on the values of *ε*_*r*2*x*_ and *ε*_*r*2*z*_ (see Supplementary Information section 1 for detailed expressions). To further evaluate this performance, let us consider an incident angle *θ*_*1*_ = 45°. The plot of the analytical expression of the out-of-plane magnetic field (H_y_) distribution before the change in *ε*_*r*_ (*t* < *t*_1_) is shown in Fig. 2b, along with the distribution of the instantaneous Poynting vector (black arrows). Now, at *t* = *t*_1_ = 38 *T*, *ε* is changed from isotropic *ε*_*r*1_ = 10 to $\overline{\overline {\varepsilon _{r2}}}$ = [*ε*_*r*2*x*_ = 8, *ε*_*r*2*z*_ = 12]. The analytical H_y_ field distribution for the FW wave for a time *t* = 38.2 *T* > *t*_1_ is shown in Fig. 2c. From these results, it can be seen how ***k*** is preserved with *θ*_1*k*_ = *θ*_2*k*_ = *θ*_*k*_ = *θ*_1_ = 45° while the instantaneous Poynting vector has an angle *θ*_*2S*_ = 56.3°, in agreement with the analytically calculated values from Fig. 2a (extracted from Eq. (2)). As shown in Fig. 2a, if one needs to further increase *θ*_2*S*_, we can just use a different value of $\overline{\overline {\varepsilon _{r2}}}$. For instance, a snapshot of the analytical H_y_ distribution of the FW wave using $\overline{\overline {\varepsilon _{r2}}}$ = [*ε*_*r*2*x*_ = 2, *ε*_*r*2*z*_ = 20] is shown in Fig. 2d at the same time instant as in Fig. 2c. As observed, *θ*_2*S*_ is further increased to 81.4° for the same initial angle *θ*_*1*_ = 45°. An animation showing this latter scenario can be seen in Supplementary Video 1.

For completeness, let us now evaluate the case where *ε*_*r*2*z*_ < *ε*_*r*2*x*_. Following the same idea as in Fig. 2a–d, *ε*_*r*_ is changed from isotropic *ε*_*r1*_ = 10 to $\overline{\overline {\varepsilon _{r2}}}$ = [*ε*_*r2x*_ = 12, *ε*_*r*2*z*_ = 8] and $\overline{\overline {\varepsilon _{r2}}}$ = [*ε*_*r*2*x*_ = 20, *ε*_*r*2*z*_ = 2] at *t* = *t*_1_ = 38 *T*. The analytically derived angles of the instantaneous Poynting vector *θ*_2*S*_ are shown in Fig. 2e as blue and black circles, respectively. By comparing these results with those shown in Fig. 2a, we notice that *θ*_*2S*_ is now larger when $\overline{\overline {\varepsilon _{r2}}}$ = [*ε*_*r*2*x*_ = 12, *ε*_*r*2*z*_ = 8] than when $\overline{\overline {\varepsilon _{r2}}}$ = [*ε*_*r*2*x*_ = 20, *ε*_*r*2*z*_ = 2], again because of the dependence of the *x* and *z* components of the electric field and *θ*_*2S*_ on the values of *ε*_*r2x*_ and *ε*_*r2z*_. For instance, if we now take the same *θ*_*1*_ = 15°, *θ*_2*S*_ will be 33.7° and 8.5° for each case. Let us now evaluate the analytically derived field distribution, and let us consider an angle *θ*_*1*_ = 65°. A snapshot of the incident H_y_ distribution for *t* < *t*_1_ is shown in Fig. 2f, along with the instantaneous Poynting vector. Now, if the relative permittivity is changed to $\overline{\overline {\varepsilon _{r2}}}$ = [*ε*_*r*2*x*_ = 12, *ε*_*r*2*z*_ = 8], the resulting H_y_ distribution at *t* = 38.2 *T* > *t*_1_ is the one shown in Fig. 2g, where we have also plotted the instantaneous Poynting vector. As observed, the angle of ***k*** is always preserved (*θ*_*1*_ = 65°), but *θ*_2*S*_ is reduced to *θ*_2*S*_ = 55°, in agreement with the results shown in Fig. 2e. If we now consider $\overline{\overline {\varepsilon _{r2}}}$ = [*ε*_*r*2*x*_ = 20, *ε*_*r*2*z*_ = 2], *θ*_2*S*_ is further reduced to *θ*_2*S*_ = 17.8°. An animation showing this latter scenario can be found in Supplementary Video 2. For the sake of completeness, snapshots in time of the BW waves for the cases shown in Fig. 2c, d and Fig. 2g, h are shown in Supplementary Information section 2.

These results demonstrate how the value of *θ*_2*S*_ will differ from the wavenumber direction *θ*_1*k*_ = *θ*_2*k*_ = *θ*_*k*_ = *θ*_1_ when *ε*_*r*_ is changed from isotropic to anisotropic values. Moreover, this *θ*_2*S*_ can be tuned to smaller/larger angles than *θ*_1*k*_ = *θ*_2*k*_ = *θ*_*k*_ = *θ*_1_ by properly engineering the values of the tensor $\overline{\overline {\varepsilon _{r2}}}$ = [*ε*_*r*2*x*_, *ε*_*r*2*z*_], a feature that is important for the proposed temporal aiming approach.

### Multiple plane waves: Gaussian beam propagation

**Fig. 3:**
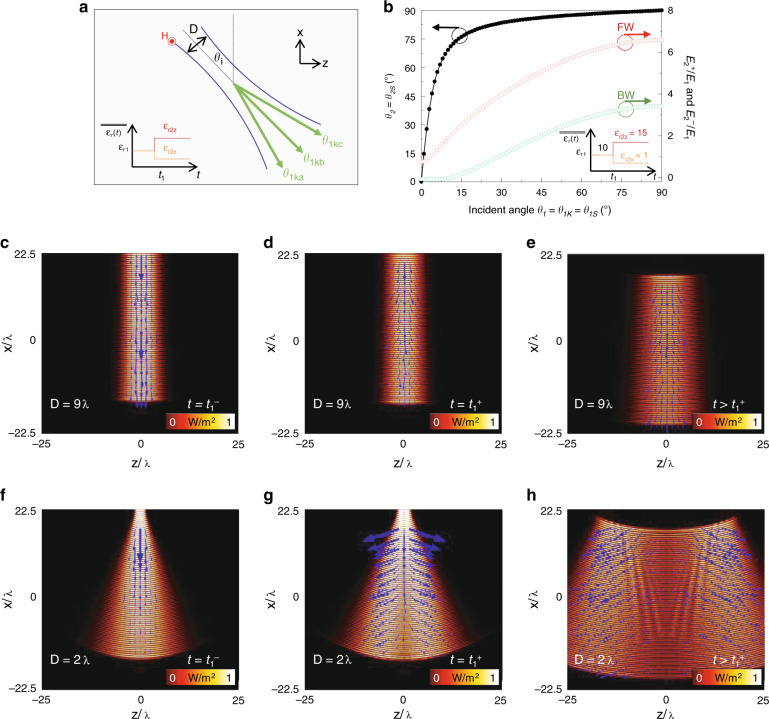
Temporal aiming with monochromatic Gaussian beams. **a** Schematic representation of an oblique incident Gaussian beam immersed in a time-dependent metamaterial. **b** Analytically derived angles of the instantaneous Poynting vector *θ*_2*s*_ (*t* > *t*_1_) as a function of the incident angle *θ*_1*k*_ (*t* < *t*_1_*)* when *ε*_*r*_ is changed from isotropic with *ε*_*r*1_ = 10 to anisotropic $\overline{\overline {\varepsilon _{r2}}}$ = [*ε*_*r*2*x*_ = 1*, ε*_*r*2*z*_ = 15] (black circles), along with the amplitude of the FW (E_2_^+^/E_1_) and BW (E_2_^−^/E_1_) electric fields (red and green circles, respectively). **c**–**e** Numerical results of the snapshot of the power-flow distribution and instantaneous Poynting vector (blue arrows) distributions for a Gaussian beam with a beam waist diameter *D* = 9*λ* before the change in *ε*_*r*_ (when *ε*_*r1*_ = 10) at *t* = 30.2 *T* (*t* = *t*_1_^−^) and after the change in the relative permittivity to $\overline{\overline {\varepsilon _{r2}}}$ = [*ε*_*r*2*x*_ = 1*, ε*_*r*2*z*_ = 15] at *t* = 30.4 *T* (*t* = *t*_1_^+^) and at *t* = 36.3 *T* (*t* > *t*_1_), respectively. **f**–**h** Numerical results of the snapshot of the power-flow distribution and instantaneous Poynting vector (blue arrows) distributions at the same times as in panels **c**–**e** and using the same time-dependent *ε*_*r*_(*t*) but considering a Gaussian beam with a beam waist diameter of *D* = 2*λ*. In all the numerical results, the incoming signal is switched off once the temporal boundary is induced at *t* = *t*_1_ to appreciate the effect of using a time-dependent *ε*_*r*_(*t*) on a signal already present in the medium

In the previous section, we evaluated the case where the relative permittivity of the medium is modified from isotropic to anisotropic values for a monochromatic CW plane wave under oblique incidence *θ*_*i*_. However, what would happen if we use more complex waves, such as a Gaussian beam? To answer this question, let us study an oblique incident monochromatic *p-*polarised Gaussian beam (in-plane *E* field), as schematically shown in Fig. 3a. As is well known, a Gaussian beam can be modelled as a summation of multiple plane waves travelling in different directions (same magnitude of the wavenumber but different directions for vector ***k*** in the same figure)^52^. Moreover, it is also known that the angular aperture of the Gaussian beam will be large/small for small/large values of the beam waist diameter *D*. If we then use a time-dependent metamaterial for the medium through which the Gaussian beam is travelling, as in the examples from Fig. 2 (i.e., *ε*_*r*_ from *ε*_*r1*_ to $\overline{\overline {\varepsilon _{r2}}}$ = [*ε*_*r*2*x*_*, ε*_*r*2*z*_]), one will expect to preserve ***k*** (in every direction) for *t* > *t*_1_, while the angle of the instantaneous Poynting vector *θ*_2*S*_ will be different for each direction of ***k***. This is because each plane wave forming the Gaussian beam will have different *θ*_1*k*_ = *θ*_2*k*_ = *θ*_*k*_ = *θ*_1_ (*θ*_1*kn*_, with *n* = a, b, c … denoting each of the multiple plane waves forming the Gaussian beam). To visualise this, let us calculate the analytical values of *θ*_*2S*_ as a function *θ*_1*k*_, as shown in Eq. (2), considering the expression for plane waves. The results are shown as black circles in Fig. 3b when relative *ε*_*r*_ is modified from *ε*_*r*1_ = 10 to $\overline{\overline {\varepsilon _{r2}}}$ = [*ε*_*r*2*x*_ = 1*, ε*_*r*2*z*_ = 15]. Note that the same trend as in Fig. 2a is observed since *ε*_*r*2*x*_ < *ε*_*r*2*z*_, showing a clear dependence of *θ*_*2S*_ on *θ*_*1k*_, as expected. To better understand these results using monochromatic Gaussian beams, let us consider an incident angle *θ*_*i*_ = 0° and a beam waist diameter *D* = 9*λ*. Here, the permittivity of the whole medium is the same as in Fig. 3b, where it is initially *ε*_*r*1_ = 10 and then it is changed to $\overline{\overline {\varepsilon _{r2}}}$ = [*ε*_*r*2*x*_ = 1*, ε*_*r*2*z*_ = 15] at *t* = *t*_1_ = 30.3 *T*. With this set-up, the numerical results of the power-flow distribution (i.e., the magnitude of the instantaneous power flow) on the *xz* plane, along with the instantaneous Poynting vector (blue arrows), for a time *t* = 30.2 *T* (*t* = *t*_1_^−^) are shown in Fig. 3c. As observed, most of the energy is travelling near *θ* = 0° because of the large beam waist diameter (*D* = 9*λ*), as expected. The power-flow distribution and the instantaneous Poynting vector for a time instant just after the change in permittivity to an anisotropic value (*t* = 30.4 *T*, *t* = *t*_1_^+^) are shown in Fig. 3d. By comparing these results with those from Fig. 3c, it can be clearly seen that the Poynting vector angles *θ*_2*S*_ are still close to *θ* = 0°, but they are indeed modified compared with *θ*_1*kn*_. These results are in agreement with the analytical angles shown in Fig. 3b, where a value of *θ*_2*S*_ = 0° will be achieved for an angle *θ*_1*k*_ = 0° and can be increased to *θ*_2*S*_ ≈ 15° for a small angle *θ*_1*k*_ = 1°. For completeness, the power-flow distribution and instantaneous Poynting vector for a time *t* > *t*_1_ are shown in Fig. 3e.

The results discussed in Fig. 3c–e were obtained considering a large beam waist diameter (*D* = 9*λ*). However, what if we use a smaller value of *D*? To evaluate this case, we use *D* = 2*λ*, and the results of the power-flow distribution and instantaneous Poynting vector for a time *t* = 30.2 *T* (before the change in *ε*_*r*_) are shown in Fig. 3f. As clearly shown, the angular aperture of the Gaussian beam is increased (as expected), and the energy is now travelling along multiple *θ*_1*kn*_ directions. With this set-up, let us now change *ε*_*r*_ to an anisotropic value, as in Fig. 3d. The results of the power-flow distribution and instantaneous Poynting vector at *t* = 30.4 *T* are shown in Fig. 3g. As observed, ***k*** is preserved, as expected, but we can now clearly see how multiple directions of *θ*_2*S*_ are obtained, in agreement with the description provided by the plane waves in Fig. 3b. The power-flow distribution and instantaneous Poynting vector for a time *t* > *t*_1_, as shown in Fig. 3e, are shown in Fig. 3h. From this figure, one can notice that the magnitude of the power-flow distribution is larger for angles other than 0°. This observation can be explained by looking at the red curve in Fig. 3b, which shows the amplitude of the electric field for the FW wave as a function of the incident angle *θ*_*1k*_ (values calculated using Eq. (1)). As observed, there is a clear dependence of E_2_^+^/E_1_ on *θ*_1*k*_, achieving an increased/reduced amplitude for large/small values of *θ*_*1k*_ when considering the values under study of $\overline{\overline {\varepsilon _{r2}}}$ = [*ε*_*r*2*x*_ = 1*, ε*_*r*2*z*_ = 15]. For instance, E_2_^+^/E_1_ is 0.74 when *θ*_1*k*_ = 0° and increases up to 6.6 when *θ*_*1k*_ = 90°. Similarly, for the BW wave (green circles in Fig. 3b), E_2_^−^/E_1_ is −0.074 when *θ*_1*k*_ = 0°; then, it reaches its maximum of 3.4 when *θ*_1*k*_ = 90°. For completeness, the amplitudes of the FW and BW waves obtained using the temporal permittivity functions studied in Fig. 2 are also shown in Supplementary Fig. S3 of the Supplementary Materials. Animations showing the examples from Fig. 3c–e and Fig. 3f–h can be found in Supplementary Movie 3.

**Fig. 4:**
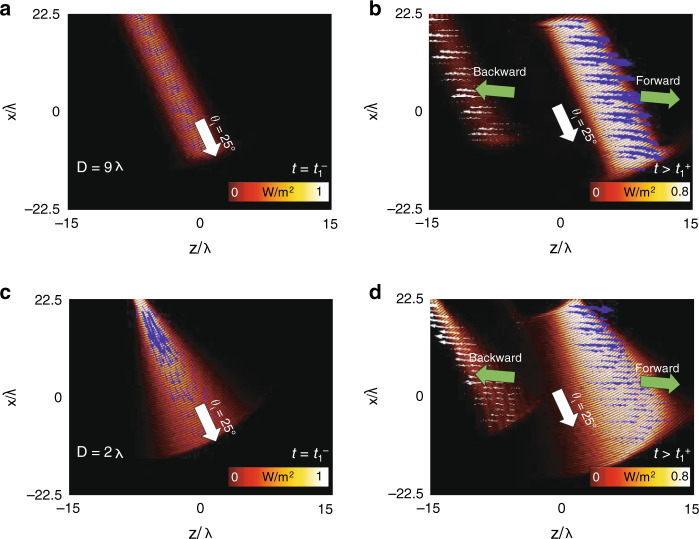
Temporal aiming with monochromatic Gaussian beams under oblique incidence. Similar to Fig. 3 but for oblique incidence. **a**, **b** Numerical results of the snapshot of the power-flow distribution and instantaneous Poynting vector (blue arrows) distributions for a Gaussian beam with a beam waist diameter *D* = 9*λ* before the change in *ε*_*r*_ (when *ε*_*r1*_ = 10) at *t* = 30.2 *T* (*t* = *t*_1_^−^) and after the change in permittivity to $\overline{\overline {\varepsilon _{r2}}}$ = [*ε*_*r*2*x*_ = 1*, ε*_*r*2*z*_ = 15] at *t* = 36.3 *T* (*t* > *t*_1_), respectively. **c**, **d** Numerical results of the snapshot of the power-flow distribution and instantaneous Poynting vector distributions using the same set-up, times and change in *ε*_*r*_ as in panels **a**, **b** but for a Gaussian beam with a beam waist diameter *D* = 2*λ*. As in Fig. 3, in all the numerical results, the incoming signal is switched off once the temporal boundary is induced at *t* = *t*_1_ to appreciate the effect of using a time-dependent *ε*_*r*_*(t*) on a signal already present in the medium. Moreover, note that the scale bars for panels **b**, **d** were saturated from 0 to 0.8 to better appreciate the FW and BW waves produced at the temporal boundary

The results discussed in Fig. 3 were calculated considering normal incident Gaussian beams at *θ*_*i*_ = 0°. For completeness, the numerical results of the power-flow distribution and instantaneous Poynting vector using oblique incident Gaussian beams with *θ*_*i*_ = 25° and the same beam waist diameters as in Fig. 3, i.e., *D* = 9*λ* and *D* = 2*λ*, are shown in Fig. 4a, b and Fig. 4c, d, respectively. As observed for a time *t* = *t*_1_^–^ (i.e., when *ε*_*r*1_ = 10), most of the energy travels along the incident angle *θ*_*i*_ = 25° for *D* = 9λ (Fig. 4a), while it spreads to more angles when *D* = 2*λ* (Fig. 4c), as expected. Now, when *ε* is changed to an anisotropic tensor $\overline{\overline {\varepsilon _{r2}}}$ = [*ε*_*r*2*x*_ = 1*, ε*_*r*2*z*_ = 15] (Fig. 4b, d), the energy is re-directed to *θ*_2*S*_, which is no longer parallel to the angle of the wavenumber ***k****, θ*_1*kn*_, as explained before. Moreover, note that in the results shown in Fig. 4b, d, the BW waves created at the temporal boundary are clearer than the results shown in Fig. 3. This is because of the dependence of the amplitude of the FW and BW waves on the angle *θ*_1*k*_. Since larger angles *θ*_1*k*_ are generated when tilting the Gaussian beam to an angle *θ*_*i*_ = 25° (compared with *θ*_*i*_ = 0° in Fig. 3), the amplitudes of both FW and BW waves are further increased (as detailed in Fig. 3b and Eq. (1)).

### Temporal aiming narrowband Gaussian wavepacket

**Fig. 5:**
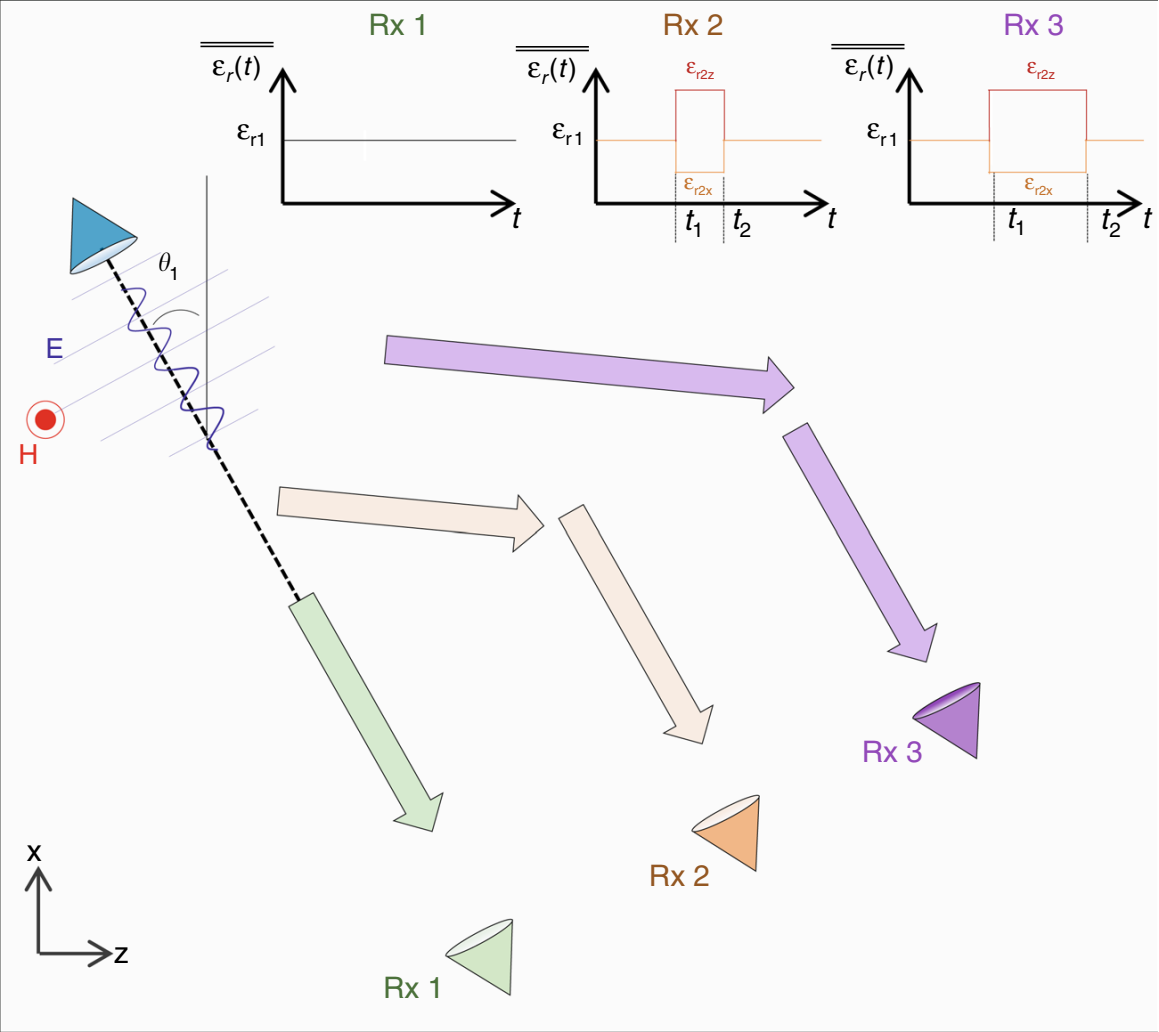
Sketch of the idea of temporal aiming with narrowband Gaussian beams under oblique incidence. The time-dependent *ε*_*r*_ functions used to redirect the wavepacket to each receiver (Rx1, Rx2 and Rx3) are shown in the insets

In the previous section, we analysed the temporal aiming approach using time-dependent metamaterials with an isotropic-to-anisotropic change in the relative *ε*_*r*_ by considering a monochromatic plane wave under oblique incidence and Gaussian beams with different beam waist diameters. In this section, we discuss how temporal aiming can be achieved when the source generates a narrowband Gaussian wavepacket. A schematic representation of this scenario is shown in Fig. 5. Let us consider an oblique incident (*θ*_1_) Gaussian wavepacket propagating in a medium with a time-dependent *ε*_*r*_ [*ε*_*r*_ (*t*), *µ*_*r*_ = 1]. Moreover, consider that we have three receivers (Rx1, Rx2 and Rx3) placed at different spatial locations. Without loss of generality, Rx1 is directly aligned with the incoming oblique incident wavepacket, while Rx2 and Rx3 are placed at different locations (see Fig. 5).

Now, if we want to send the wavepacket to Rx1, we need only to keep *ε*(*t*) = *ε*_1_ = constant since both the source and Rx1 are aligned. However, if the wavepacket is already propagating in the medium, would it be possible to redirect it to reach either Rx2 or Rx3? In the previous section, it was shown how the Poynting vector ***S*** is modified to an angle different from the wavenumber ***k*** once the relative permittivity is changed from an isotropic value to an anisotropic tensor. Based on this, a way to deflect the propagating wavepacket and reach Rx2, for instance, would be to engineer a time-dependent *ε*(*t*), as shown in the inset of Fig. 5 for this receiver. In this case, *ε*_*r*_ can be changed from *ε*_*r*1_ to $\overline{\overline {\varepsilon _{r2}}}$ = [*ε*_*r*2*x*_ ≠ *ε*_*r*2*z*_] at *t* = *t*_1_ and kept at this value for a certain time duration Δ*t* = *t*_2_–*t*_1_. During this time interval, the wavepacket preserves ***k*** (as explained in the previous sections), but the direction of the energy flow (***S***) changes. Hence, the interval Δ*t* should be selected such that the wavepacket moves along the *xz* plane until it is aligned with the receiver Rx2 at time *t* = *t*_2_^−^. Once this step is achieved, then ε_*r*_ can be returned from $\overline{\overline {\varepsilon _{r2}}}$ = [*ε*_*r*2*x*_ ≠ *ε*_*r*2*z*_] to its original value *ε*_*r*1_ at *t* = *t*_2_ to allow the wavepacket to travel again with the initial angle *θ*_*1*_, reaching Rx2. Similarly, this process can be performed to redirect the pulse to Rx3, and the same values of *ε*_*r*1_ and $\overline{\overline {\varepsilon _{r2}}}$ = [*ε*_*r*2*x*_ ≠ *ε*_*r*2*z*_] can be applied as in the previous case. The only difference would be that the time interval Δt should now be modified accordingly to align the wavepacket to this receiver.

**Fig. 6:**
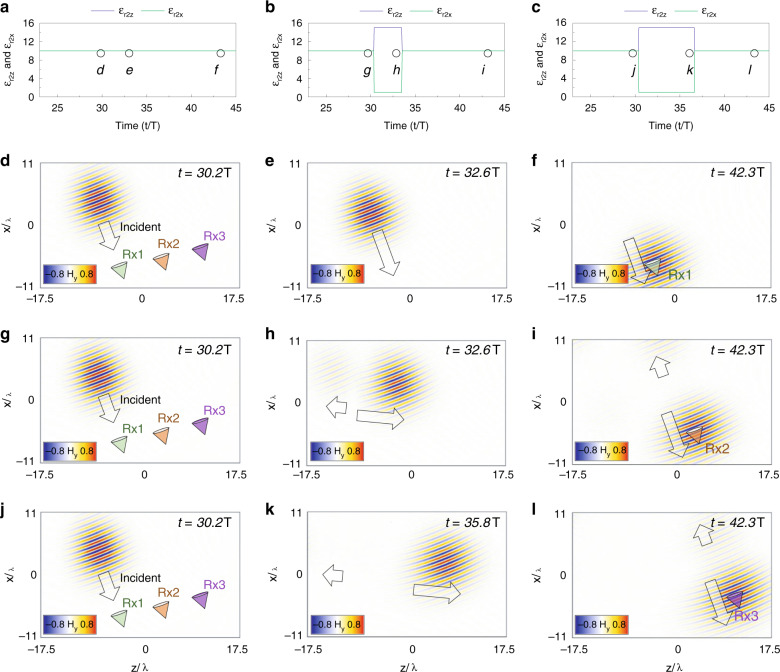
Results of temporal aiming with narrowband Gaussian wavepacket under oblique incidence. **a**–**c** Time-dependent *ε*_*r*_ to redirect an oblique incident (*θ*_*1*_ = 25°) narrowband wavepacket towards each receiver Rx1, Rx2 and Rx3, respectively. **d**–**f** Snapshot of the H_y_ field distribution at times *t* < *t*_1_, *t*_1_ < *t* < *t*_2_ and *t* > *t*_2_, respectively, considering the time-dependent *ε*_*r*_ shown in panel **a**. **g**–**i** Snapshot of the H_y_ field distribution at times *t* < *t*_1_, *t*_1_ < *t* < *t*_2_ and *t* > *t*_2_, respectively, considering the time-dependent ε shown in panel **b**. **j**–**l** Snapshot of the H_y_ field distribution at times *t* < *t*_1_, *t*_1_ < *t* < *t*_2_ and *t* > *t*_2_, respectively, considering the time-dependent *ε*_*r*_ shown in panel **c**. The times at which panels **d**–**l** have been obtained are shown as circles in panels **a**–**c** to guide the eye

An example of the temporal aiming described in Fig. 5 is presented in Fig. 6 using an oblique incident narrowband wavepacket with *θ*_*1*_ = 25°. The specific time-dependent *ε*_*r*_(*t*) of the medium for re-directing the wavepacket to Rx1, Rx2 and Rx3 is shown in Fig. 6a–c, respectively. For Rx1 (Fig. 6a), the permittivity ε_r_ is constant at *ε*_*r*1_ = 10, with no change because the source is aligned to this receiver, as explained before. The numerical results of the H_y_ field distribution at different times for this case are shown in Fig. 6d–f, where it can be seen how the pulse is directly sent to Rx1. Now, to reach Rx2, the relative ε_r_ is changed from isotropic *ε*_*r*1_ = 10 to anisotropic $\overline{\overline {\varepsilon _{r2}}}$ = [*ε*_*r*2*x*_ = 1*, ε*_*r*2*z*_ = 15] (the same values as in Fig. 4) at *t* = *t*_1_ = 30.3 *T* and kept at this value until it is returned to ε_r1_ = 10 at *t* = *t*_2_ = 33.5 *T* (Δ*t* = 3.2 *T*). The numerical results of the H_y_ field distribution for a time *t* < *t*_1_, *t*_1_ < *t* < *t*_2_ and *t* > *t*_2_ for this case are shown in Fig. 6g–i, respectively. As observed, when *t*_1_ < *t* < *t*_2_ (Fig. 6h), the wavepacket propagates with an angle defined by the Poynting vector (*θ*_*2S*_ ≈ 82°, in agreement with the analytical values from Eq. (2), which predicts *θ*_2*S*_ = 81.4°). Finally, once ε is returned to the isotropic state with *ε*_*r*1_ = 10, the wavepacket propagates with the same incident angle (*θ*_*1*_ = 25°) and is able to reach Rx2 (Fig. 6i). The same process is then applied to the wavepacket to reach Rx3, but now the parameter Δ*t* is increased to Δ*t* = 6.3 *T*. The ε_r_ function for this receiver and the H_y_ field distribution at different times are shown in Fig. 6c, j–l, demonstrating how the wavepacket can reach Rx3 using this time-dependent function of *ε*_*r*_. An animation showing the results of the temporal aiming described in Fig. 6 can be found in Supplementary Video 4. Finally, it is important to note that we change here the relative permittivity of the whole medium through which the wave is travelling. This temporal aiming may be achieved using 2D transmission lines loaded with time-varying circuit elements or with tuneable metasurfaces^36,53^.

## Discussion

In conclusion, we have discussed time-dependent metamaterials by inducing temporal boundaries using a rapid change in permittivity from isotropic to anisotropic values. The physics behind this temporal variation of the electromagnetic properties of the medium has been presented, highlighting that the wavenumber and instantaneous Poynting vector of the electromagnetic waves already propagating in this medium exhibit different directions determined by the change in the permittivity tensor. This performance has been exploited to achieve *temporal aiming*, where an electromagnetic wavepacket can be ushered and re-directed to desired angles by engineering the isotropic–anisotropic temporal variation of the relative permittivity. Different examples have been evaluated both numerically and analytically, such as plane waves under oblique incidence and monochromatic normal and oblique incident Gaussian beams. Moreover, our *temporal aiming* approach was also evaluated considering the case of an oblique incident narrowband Gaussian wavepacket, showing that it can be possible to deflect and redirect a wavepacket to reach receivers placed at different spatial locations by using isotropic–anisotropic–isotropic temporal metamaterials. The ideas presented here may find applications in integrated photonics scenarios where it may be required to redirect and send waves to specific targets/receivers on photonic chips in real time, and may open up new avenues in manipulating waves by ushering and guiding wavepackets at will.

## Materials and methods

All numerical simulations were performed using the time-domain solver of the commercial software COMSOL Multiphysics^®^. For all the simulations, a rectangular box of dimensions 105*λ* × 62.5*λ* was implemented. The incident field was applied from the top boundary of the simulation box via a scattering boundary condition with an out-of-plane magnetic field. The complete non-paraxial Gaussian beam expression was introduced using the angular spectrum technique for plane waves. In this method, the Gaussian beam is calculated as a summation (integral in our case) of multiple plane waves propagating with the same magnitude of wavenumber ***k*** but in different directions^52^. Scattering boundary conditions were also implemented on the bottom, left and right boundaries of the simulation box to avoid undesirable reflections. Finally, a triangular mesh was implemented with minimum and maximum sizes of 1.5 × 10^−8^*λ* and 0.1 *λ*, respectively, to ensure accurate results. The rapid changes in ε in all the studies were modelled by implementing rectangular analytical functions with smooth transitions using two continuous derivatives to ensure convergence in the calculations.

## Supplementary information

Supplementary information

video 1

video 2

video 3

video 4
