# Supplementary material for: Temporal aiming
Source: Light Sci Appl. 2020 Jul 20;9:129. doi: 10.1038/s41377-020-00360-1 (PMC7371637; doi:10.1038/s41377-020-00360-1)
Supplement: Supplementary file 1 — Supplementary information [file 41377_2020_360_MOESM1_ESM.docx]

**Supplementary information**

***Temporal aiming***

Victor Pacheco-Peña^1^ and Nader Engheta^2*^

^1^School of Mathematics, Statistics and Physics, Newcastle University, Newcastle Upon Tyne, NE1 7RU, United Kingdom

^2^Department of Electrical and Systems Engineering, University of Pennsylvania, Philadelphia, PA 19104, USA

^*^email: [*Victor.Pacheco-Pena@newcastle.ac.uk*](mailto:Victor.Pacheco-Pena@newcastle.ac.uk)*,*  [engheta@ee.upenn.edu](mailto:engheta@ee.upenn.edu)

1. **Analytical formulation: temporal boundary isotropic–to- anisotropic ε_r_(t).**
2. **Analytical BW waves examples: isotropic–to-anisotropic temporal relative permittivity.**
3. **Received field distribution in time: temporal aiming with three receivers**
4. **Index of Supplementary Movies.**
5. **Analytical formulation: temporal boundary isotropic–to-anisotropic ε_r_(t).**

In this section, for the sake of completeness and easy access we provide the analytical formulation considering the case where a temporal boundary is induced by changing the relative permittivity (of positive value greater than or equal to unity) in time from isotropic to anisotropic values (with each tensor element to be equal to or greater than unity). The aim here is to show how the instantaneous Poynting vector is modified to a different direction compared to the wave vector when introducing such change of the relative permittivity tensor.

To begin with, the schematic representation of the problem under study is shown in Fig. S1. A monochromatic plane wave with an in-plane E-field (TM polarization) is traveling in an unbounded medium with an angle *θ_1_* as shown. Initially for t < t_1_ the relative permittivity of the whole medium is isotropic (*ε_r1_* *= ε_r1x_* *= ε_r1z_*) and it is rapidly changed to anisotropic values at t = t_1_ to$\varepsilon_{r2}$= {*ε_r2x_, ε_r2z_*} with *ε_r2x_ ≠ ε_r2z_*. Note that here we only consider the *z* and *x* components of the relative permittivity tensor since we have a TM polarization with the electric field lying on this *xz* plane. Similarly, and for the sake of completeness, we can consider that the relative permeability is also changed from *µ_r1_* = *µ_r1y_* for t < t_1_ the to *µ_r2_ = µ_r2y_* at t = t_1_, again taking into account the TM polarization of the incident monochromatic planewave.


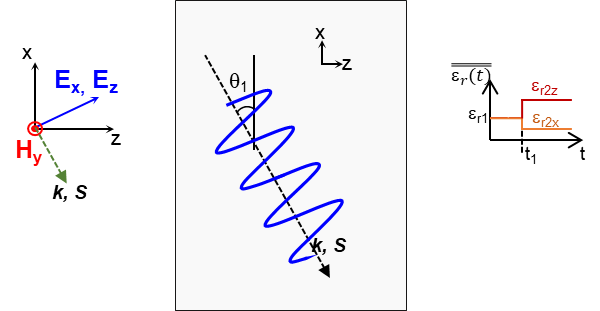


**Figure S1| Schematic representation of the problem under study.** A monochromatic plane wave traveling with an angle *θ_1_* in a medium that has a time dependent relative permittivity tensor that is changed from isotropic to anisotropic values.

With this setup, let us first define the E and H fields before the anisotropic temporal boundary (t < t_1_), i.e. when the permittivity is isotropic, as follows:

$H_{1}=\hat{y}e^{i\left( {}_{1}t-k_{x}x-k_{z}z \right)}$ (S.1)

$E_{1}=\frac{1}{\varepsilon_{0}{}_{1}\varepsilon_{r1}}e^{i\left( {}_{1}t-k_{x}x-k_{z}z \right)}\left[ \begin{matrix} k_{z} \\ 0 \\ -k_{x} \end{matrix} \right]$ (S.2)

where ω_1_ = 2π*f_1_*, *k_x_ = -k cos(θ_1_), k_z_ = k sin(θ_1_),* $k={{}_{1}}/{v_{1}}$ is the wave number, $v_{1}=c/{\sqrt{{}_{r1}\varepsilon_{r1}}}$ and *c* is the velocity of light in vacuum. Now, at t = t_1_ the relative permittivity of the whole medium is rapidly changed from isotropic (*ε_r1_* *= ε_r1x_* *= ε_r1z,_ µ_r1y_*) to an anisotropic relative permittivity tensor $\varepsilon_{r2}$= [*ε_r2x_, ε_r1z_*] and permeability *µ_r2y_*. In this scenario, the induced temporal boundary will produce a set of a forward (FW, E^+^) and a backward (BW, E^-^) waves. These two new created waves are the temporal equivalent to transmitted and reflected waves produced at a spatial interface between two materials of different electromagnetic parameters^1,2^. With this configuration, the total magnetic and electric fields for t > t_1_ in the unbounded medium can be defined as follows:

$H_{2}=H_{2}^{+}-H_{2}^{-}$ (S.3a)

$H_{2}=\hat{y}e^{i\left( -k_{x}x-k_{z}z \right)}\left\{ Ae^{i\left[ {}_{2}\left( t-t_{1} \right) \right]}-Be^{-i\left[ {}_{2}\left( t-t_{1} \right) \right]} \right\}$ (S.3b)

$E_{2}=E_{2}^{+}+E_{2}^{-}$ (S.4a)

$E_{2}=\frac{1}{\varepsilon_{0}{}_{2}}\left( \hat{x}\frac{k_{z}}{\varepsilon_{r2x}}-\hat{z}\frac{k_{x}}{\varepsilon_{r2z}} \right)e^{i\left( -k_{x}x-k_{z}z \right)}\left\{ Ae^{i\left[ {}_{2}\left( t-t_{1} \right) \right]}+Be^{-i\left[ {}_{2}\left( t-t_{1} \right) \right]} \right\}$ (S.4b)

With

${}_{2}==c\sqrt{\frac{k_{x}^{2}}{\varepsilon_{2z}\mu_{2y}}+\frac{k_{z}^{2}}{\varepsilon_{2x}\mu_{2y}}}$ (S.5)

The superscripts +/- in Eqs. S.3a and S.4a correspond to the forward and backward waves, respectively. The next step is to calculate the constants A and B by applying the conservation of **D** and **B** at t = t_1_, i.e., **D_t1-δ_** = **D_t1+δ_** and **B_t1-δ_** = **B_t1+δ_** in the limit when δ→0, as follows^1–3^:

Conservation of **B**:

$B_{1}=B_{2}$ (S.6a)

$\frac{\mu_{r1}}{\mu_{r2y}}e^{i\left( {}_{1}t_{1} \right)}=A-B$ (S.6b)

Conservation of **D**:

$D_{1}=D_{2}$ (S.7a)

$\frac{{}_{2}}{{}_{1}}e^{i\left( {}_{1}t_{1} \right)}=A+B$ (S.7b)

After working with Eqs. S.6-S.7, the constants A and B are defined as:

$A=\frac{1}{2}\left[ \frac{{}_{2}}{{}_{1}}+\frac{\mu_{r1}}{\mu_{2ry}} \right]e^{i\left( {}_{1}t_{1} \right)}$ (S.8a)

$B=\frac{1}{2}\left[ \frac{{}_{2}}{{}_{1}}-\frac{\mu_{1}}{\mu_{r2y}} \right]e^{i\left( {}_{1}t_{1} \right)}$ (S.8b)

Finally, the full analytical expressions for the magnetic and electric field after inducing the anisotropic temporal boundary (Eqs. S.3-S.4) are the following:

Magnetic field:

$H_{2}=H_{2}^{+}-H_{2}^{-}$ (S.9a)

$H_{2}^{+}=\hat{y}e^{i\left( -k_{x}x-k_{z}z \right)}\left\{ \frac{1}{2}\left[ \frac{{}_{2}}{{}_{1}}+\frac{\mu_{r1}}{\mu_{r2y}} \right]e^{i\left( {}_{1}t_{1} \right)}e^{i\left[ {}_{2}\left( t-t_{1} \right) \right]} \right\}$ (S.9b)

$H_{2}^{-}=-\hat{y}e^{i\left( -k_{x}x-k_{z}z \right)}\left\{ \frac{1}{2}\left[ \frac{{}_{2}}{{}_{1}}-\frac{\mu_{r1}}{\mu_{r2y}} \right]e^{i\left( {}_{1}t_{1} \right)}e^{-i\left[ {}_{2}\left( t-t_{1} \right) \right]} \right\}$ (S.9c)

Electric field:

$E_{2}=E_{2}^{+}+E_{2}^{-}$ (S.10a)

$E_{2x}^{+}=\frac{1}{\varepsilon_{0}{}_{2}}\left( \hat{x}\frac{k_{z}}{\varepsilon_{r2x}} \right)e^{i\left( -k_{x}x-k_{z}z \right)}\left\{ \frac{1}{2}\left[ \frac{{}_{2}}{{}_{1}}+\frac{\mu_{r1}}{\mu_{r2y}} \right]e^{i\left( {}_{1}t_{1} \right)}e^{i\left[ {}_{2}\left( t-t_{1} \right) \right]} \right\}$ (S.10b)

$E_{2x}^{-}=\frac{1}{\varepsilon_{0}{}_{2}}\left( \hat{x}\frac{k_{z}}{\varepsilon_{r2x}} \right)e^{i\left( -k_{x}x-k_{z}z \right)}\left\{ \frac{1}{2}\left[ \frac{{}_{2}}{{}_{1}}-\frac{\mu_{r1}}{\mu_{r2y}} \right]e^{i\left( {}_{1}t_{1} \right)}e^{-i\left[ {}_{2}\left( t-t_{1} \right) \right]} \right\}$ (S.10c)

$E_{2z}^{+}=-\frac{1}{\varepsilon_{0}{}_{2}}\left( \hat{z}\frac{k_{x}}{\varepsilon_{r2z}} \right)e^{i\left( -k_{x}x-k_{z}z \right)}\left\{ \frac{1}{2}\left[ \frac{{}_{2}}{{}_{1}}+\frac{\mu_{r1}}{\mu_{r2y}} \right]e^{i\left( {}_{1}t_{1} \right)}e^{i\left[ {}_{2}\left( t-t_{1} \right) \right]} \right\}$ (S.10d)

$E_{2z}^{-}=-\frac{1}{\varepsilon_{0}{}_{2}}\left( \hat{z}\frac{k_{x}}{\varepsilon_{r2z}} \right)e^{i\left( -k_{x}x-k_{z}z \right)}\left\{ \frac{1}{2}\left[ \frac{{}_{2}}{{}_{1}}-\frac{\mu_{r1}}{\mu_{r2y}} \right]e^{i\left( {}_{1}t_{1} \right)}e^{-i\left[ {}_{2}\left( t-t_{1} \right) \right]} \right\}$ (S.10e)

Finally, the normalized amplitude of the electric field for the forward and backward waves can be straightforwardly calculated using Eqs. S.10 and they mathematical expressions are the following:

$\frac{E_{2}^{+}}{E_{1}}=\frac{1}{2}$ $\left[ \frac{\mu_{r2y}{}_{2}+{\mu_{r1}}_{1}}{\mu_{r2y}{}_{2}} \right]\frac{\varepsilon_{r1}\sqrt{\varepsilon_{r2z}^{2}k_{z}^{2}+\varepsilon_{r2x}^{2}k_{x}^{2}}}{\varepsilon_{r2x}\varepsilon_{r2z}\sqrt{k_{z}^{2}+k_{x}^{2}}}$ (S.11)

$\frac{E_{2}^{-}}{E_{1}}=\frac{1}{2}$ $\left[ \frac{\mu_{r2y}{}_{2}-{\mu_{r1}}_{1}}{\mu_{r2y}{}_{2}} \right]\frac{\varepsilon_{r1}\sqrt{\varepsilon_{r2z}^{2}k_{z}^{2}+\varepsilon_{r2x}^{2}k_{x}^{2}}}{\varepsilon_{r2x}\varepsilon_{r2z}\sqrt{k_{z}^{2}+k_{x}^{2}}}$ (S.12)

Note that, if µ_r2y_ = µ_1_, i.e., the amplitudes converge to the case where only the relative permittivity is changed in time^3^. Moreover, if the change of relative permittivity is from isotropic (ε_r1_) to another positive isotropic value (ε_r2_ = ε_r2x_ = ε_r2z_) Eqs. S.11-S.12 converge to:

$\frac{E_{2}^{+}}{E_{1}}=\frac{1}{2}$ $\left[ \frac{\varepsilon_{r1}}{\varepsilon_{r2}}+\frac{\sqrt{\mu_{r1}\varepsilon_{r1}}}{\sqrt{\mu_{r2}\varepsilon_{r2}}} \right]$ (S.13)

$\frac{E_{2}^{-}}{E_{1}}=\frac{1}{2}$ $\left[ \frac{\varepsilon_{r1}}{\varepsilon_{r2}}-\frac{\sqrt{\mu_{r1}\varepsilon_{r1}}}{\sqrt{\mu_{r2}\varepsilon_{r2}}} \right]$ (S.14)

which are the known expressions for temporal boundaries using isotropic–to-isotropic changes of permittivity/permeability^1^.

Now, by looking at the expressions of the electric field for times t > t_1_ (Eq. 10) it is interesting to note how the wave vector *k* is preserved after the temporal boundary, as expected. Hence, *k* will preserve its direction defined by the incident angle *θ_1k_ = θ_2k_ = θ_1_*. However, the angle of the Poynting vector *S* for the same times (t > t_1_) can be calculated as:

$\theta_{2S}=\theta_{SFW}=\theta_{SBW}={tan}^{-1}\left( -\frac{E_{2x}^{+}}{E_{2z}^{+}} \right)={tan}^{-1}\left( -\frac{E_{2x}^{-}}{E_{2z}^{-}} \right)$ (S.15)

As observed above, $\theta_{2S}$is the same for both FW and BW waves. By using Eq. S.15 along with Eqs. S.10, the angle of the Poynting vector for both the FW and BW waves is the following:

$\theta_{2S}={tan}^{-1}\left( \frac{\frac{1}{\varepsilon_{0}{}_{2}}\left( \frac{k_{z}}{\varepsilon_{r2x}} \right)e^{i\left( -k_{x}x-k_{z}z \right)}\left\{ \frac{1}{2}\left[ \frac{{}_{2}}{{}_{1}}\pm\frac{\mu_{r1}}{\mu_{r2y}} \right]e^{i\left( {}_{1}t_{1} \right)}e^{\pm i\left[ {}_{2}\left( t-t_{1} \right) \right]} \right\}}{\frac{1}{\varepsilon_{0}{}_{2}}\left( \frac{k_{x}}{\varepsilon_{r2z}} \right)e^{i\left( -k_{x}x-k_{z}z \right)}\left\{ \frac{1}{2}\left[ \frac{{}_{2}}{{}_{1}}\pm\frac{\mu_{r1}}{\mu_{r2y}} \right]e^{i\left( {}_{1}t_{1} \right)}e^{\pm i\left[ {}_{2}\left( t-t_{1} \right) \right]} \right\}} \right)$ (S.16)

where the + and – inside the square brackets and the exponentials represents the FW and BW waves, respectively. Finally, Eq. S.16 can be reduced to the following simple expression:

$\theta_{2S}={tan}^{-1}\left[ \tan\left( \theta_{1} \right)\left( \frac{\varepsilon_{r2z}}{\varepsilon_{r2x}} \right) \right]$ (S.17)

retrieving the expression shown in the manuscript. As observed, the vectors ***S*** and ***k*** are pointed to different angles when modifying the relative permittivity in time from isotropic to anisotropic values and the former angle will depend on the angle of the incident wave before the temporal change (*θ_1_*) and the values of the relative permittivity tensor ε_r2x_ and ε_r2z_.

1. **Analytical BW waves examples: isotropic-to–anisotropic temporal relative permittivity**

In Fig. 2 of the main manuscript, we discussed the FW waves produced when using different isotropic–to-anisotropic changes of relative permittivity under different incident angles of the monochromatic plane wave. The corresponding field distributions of the BW waves for the same examples provided in Figure 2 are shown here in Fig. S2 for completeness. Moreover, the amplitude of the FW and BW waves as a function of the incident angle for the same examples are shown in Fig. S3.


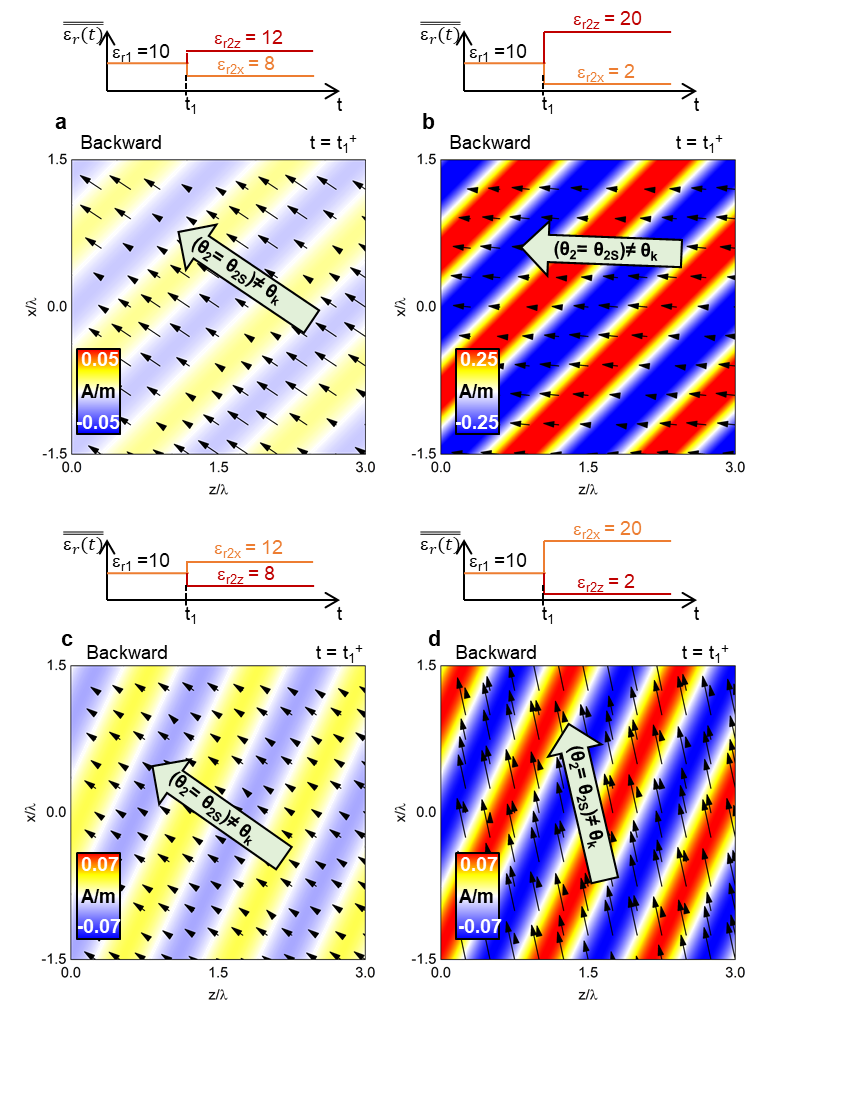


**Figure S2| Analytically derived Hy field distribution for BW wave generated when the permittivity is changed from isotropic to anisotropic values under different configurations. a. relative** ε_r_ is modified from ε_r1_ = 10 to (ε_r2z_ = 12, ε_r1x_ = 8) with *θ_1_* = 25^o^. **b.** ε_r1_ = 10 to (ε_r2z_ = 20, ε_r1x_ = 2) with *θ_1_* = 25^o^. **c.** ε_r_ is modified from ε_r1_ = 10 to (ε_r2z_ = 8, ε_r1x_ = 12) with *θ_1_* = 65^o^. **c.** ε is modified from ε_r1_ = 10 to (ε_r2z_ = 2, ε_r1x_ = 20) with *θ_1_* = 65^o^. The time-dependent permittivity tensor for these examples is schematically shown on top of each panel to guide the eye.


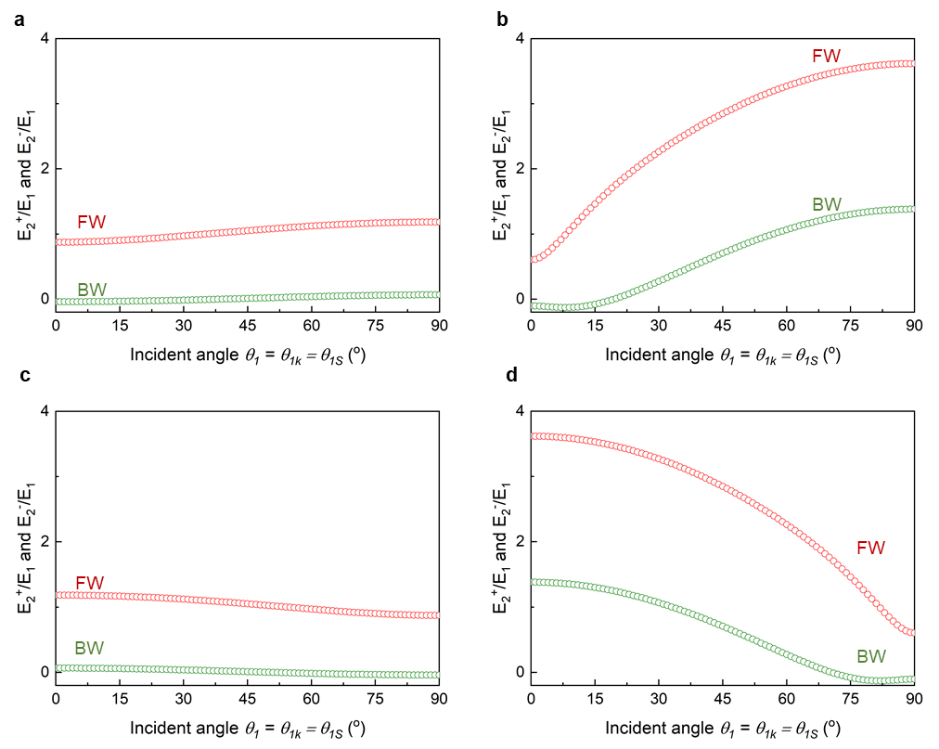


**Figure S3| Amplitude of FW and BW waves.** Analytically-derived amplitude of the FW (E_2_^+^/E_1_) and BW (E_2_^-^/E_1_) electric field (red and green circles, respectively) as a function of the incident angle *θ_1k_* (t < t_1_) when *ε_r_* is changed from isotropic with *ε_r1_* = 10 to anisotropic a, $\varepsilon_{r2}$= [*ε_r2x_* = 8*, ε_r2z_*= 12], b, $\varepsilon_{r2}$= [*ε_r2x_* = 2*, ε_r2z_*= 20], c, $\varepsilon_{r2}$= [*ε_r2x_* = 12*, ε_r2z_*= 8] and d, $\varepsilon_{r2}$= [*ε_r2x_* = 20*, ε_r2z_*= 2]. Note that these permittivity tensors correspond to the values used in Fig. 2 of the main text and Fig. S2 of this Supplementary information document.

1. **Received field distribution in time: temporal aiming with three receivers**

In Fig. 5-6 of the main manuscript, it was shown how the isotropic-to-anisotropic temporal relative permittivity tensor can be applied for temporal aiming using receivers at different spatial locations. From those results, we record the numerical values of the simulation results for the magnetic field (*H_y_*) at the location of each receiver as shown in the left column of Fig. S4. The corresponding spectral response is shown in the right column from the same figure. As observed, the incident narrow band pulse is clearly recorded by each receiver demonstrating the ability of temporal aiming using time-dependent metamaterials with isotropic-to–anisotropic changes of relative permittivity.


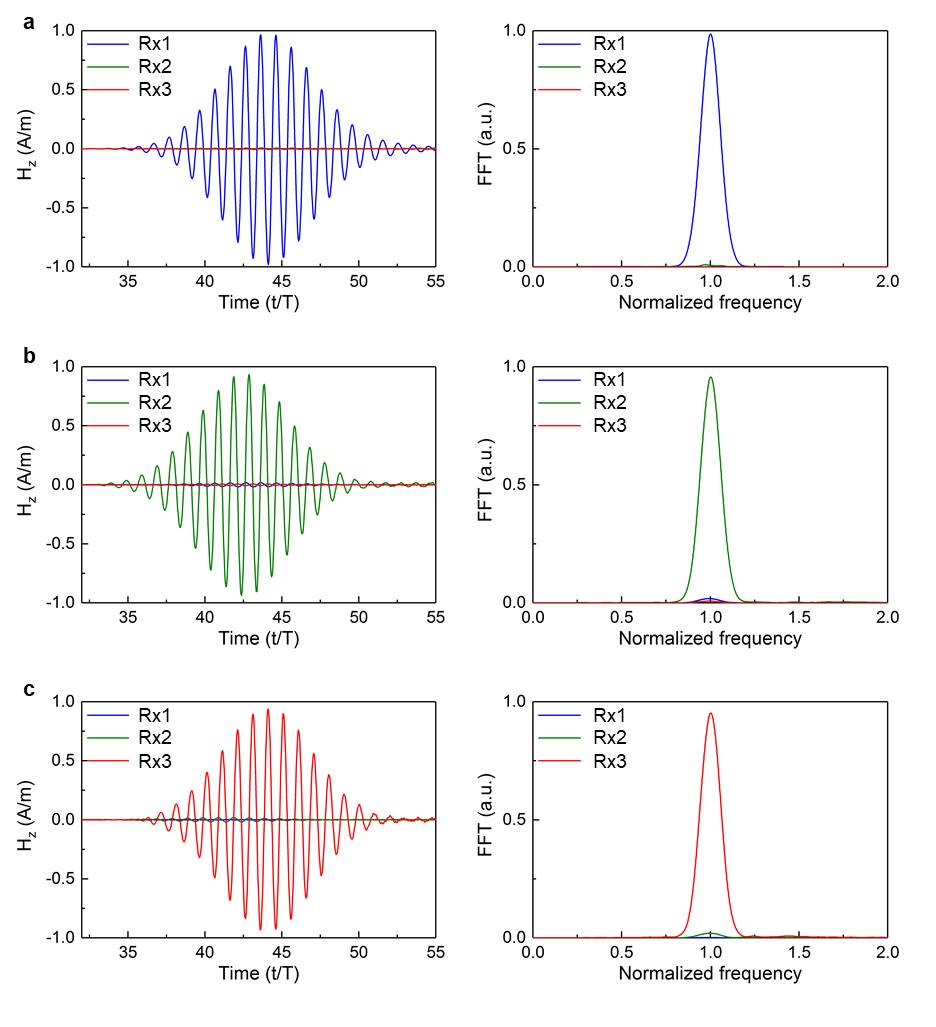


**Figure S4| Received H_y_ field at the location of each receiver. a.** Numerical values of the simulation results received at the location of Rx1, **b.** Received at the location of Rx2 and **c.** Received at the location of Rx3. The temporal signal along with the corresponding spectral response are shown in the left and right columns, respectively.

1. **Index of Supplementary Movies**

Supplementary Movie 1: Isotropic-Anisotropic temporal relative permittivity planewave oblique incidence θ_1_ = 45°.

Supplementary Movie 2: Isotropic-Anisotropic temporal relative permittivity planewave oblique incidence θ_1_ = 65°.

Supplementary Movie 3: Isotropic-Anisotropic temporal relative permittivity monochromatic Gaussian beams.

Supplementary Movie 4: Temporal aiming with three receivers.

**References:**

1. Morgenthaler, F. Velocity Modulation of Electromagnetic Waves. *IRE Trans. Microw. Theory Tech.* 167–172 (1958).

2. Fante, R. I. Transmission of Electromagnetic Waves into Time-Varying media. *IEEE Trans. Antennas Propag.* 417–424 (1971).

3. Akbarzadeh, A., Chamanara, N. & Caloz, C. Inverse Prism based on Temporal Discontinuity and Spatial Dispersion. *Opt. Lett.* **43**, 3297–3300 (2018).
